# Induction of Defense Gene Expression and the Resistance of Date Palm to *Fusarium oxysporum* f. sp. *Albedinis* in Response to Alginate Extracted from *Bifurcaria bifurcata*

**DOI:** 10.3390/md20020088

**Published:** 2022-01-20

**Authors:** Soukaina Bouissil, Claire Guérin, Jane Roche, Pascal Dubessay, Zainab El Alaoui-Talibi, Guillaume Pierre, Philippe Michaud, Said Mouzeyar, Cédric Delattre, Cherkaoui El Modafar

**Affiliations:** 1 Centre d’Agrobiotechnologie et Bioingénierie, Unité de Recherche Labellisée CNRST (Centre AgroBiotech-URL-CNRST-05), Faculté des Sciences et Techniques Marrakech, Université Cadi Ayyad, Marrakech 40000, Morocco; zainab.elalaouitalibi@gmail.com (Z.E.A.-T.); elmodafar@uca.ac.ma (C.E.M.); 2 Université Clermont Auvergne, Clermont Auvergne INP, CNRS, Institut Pascal, 63000 Clermont-Ferrand, France; pascal.dubessay@uca.fr (P.D.); guillaume.pierre@uca.fr (G.P.); cedric.delattre@uca.fr (C.D.); 3 UMR 1095 GDEC INRA-Université Clermont-Auvergne, 1 Impasse Amélie Murat, 63178 Aubière, France; guerin-cla@orange.fr (C.G.); jane.roche@uca.fr (J.R.); Said.MOUZEYAR@uca.fr (S.M.); 4 Institut Universitaire de France (IUF), 1 Rue Descartes, 75005 Paris, France

**Keywords:** alginate, date palm, elicitor, defense genes, resistance, *Fusarium oxysporum* f. sp. *albedinis*

## Abstract

In many African countries, the Bayoud is a common disease spread involving the fungus *Fusarium oxusporum* f. sp. *albedinis* (Foa). The induction of plant natural defenses through the use of seaweed polysaccharides to help plants against pathogens is currently a biological and ecological approach that is gaining more and more importance. In the present study, we used alginate, a natural polysaccharide extracted from a brown algae *Bifurcaria bifurcata*, to activate date palm defenses, which involve phenylalanine ammonia-lyase (PAL), a key enzyme of phenylpropanoid metabolism. The results obtained showed that at low concentration (1 g·L^−1^), alginate stimulated PAL activity in date palm roots 5 times more compared to the negative control (water-treated) after 24 h following treatment and 2.5 times more compared to the laminarin used as a positive stimulator of plant natural defenses (positive control of induction). Using qRT-PCR, the expression of a selection of genes involved in three different levels of defense mechanisms known to be involved in response to biotic stresses were investigated. The results showed that, generally, the PAL gene tested and the genes encoding enzymes involved in early oxidative events (SOD and LOX) were overexpressed in the alginate-treated plants compared to their levels in the positive and negative controls. POD and PR protein genes selected encoding *β*-(1,3)-glucanases and chitinases in this study did not show any significant difference between treatments; suggesting that other genes encoding POD and PR proteins that were not selected may be involved. After 17 weeks following the inoculation of the plants with the pathogen Foa, treatment with alginate reduced the mortality rate by up to 80% compared to the rate in control plants (non-elicited) and plants pretreated with laminarin, which agrees with the induction of defense gene expression and the stimulation of natural defenses in date palm with alginate after 24 h. These results open promising prospects for the use of alginate in agriculture as an inducer that triggers immunity of plants against telluric pathogens in general and of date palm against *Fusarium oxysporum* f. sp. *albedinis* in particular.

## 1. Introduction

Phoenicultural activity in Morocco has utmost economic importance in desert areas and provides, in addition to dates, the creation of a typical environment beneficial to the cultivation of other underlying crops (arboriculture, market gardening, etc.), thus guaranteeing a certain economic autonomy for the population of the Saharan oases in southern Morocco. However Bayoud disease, caused by *Fusarium oxysporum* f. sp. *albedinis* (Foa), represents a limiting factor for date palm cultivation in Morocco and constitutes a serious threat for other phoenicultural countries [1]. It was first discovered in southern Morocco in 1870, in the Daraa valley north of Zagora [2], and subsequently spread to the east and west and affected two-thirds of palm groves (10 million palm trees) in a century. Moreover, the exacerbation of Bayoud disease caused the desertification and disappearance of the underlying crops, leading to an imbalance of the oasis ecosystem [3]. The severity of this disease lies mostly in the nature of its causative agent, Foa. Foa is a soil-borne parasite that infects plant vessels and produces chlamydospores, which can survive in plant vessels and deeply in the soil [1]. Indeed, the studies carried out on the genetic diversity of Foa isolates from different stations have shown that they have a single clonal origin [4,5,6,7,8,9]. Within the framework of the fight against this pathogen, measures have been taken mainly to limit its spread by adopting a set of agricultural techniques [10]. The means of prophylactic control are ineffective due to the contamination of several palm groves and their unsustainable impact. Thus, chemical treatment through the use of systemic fungicides has been inefficient and led, thanks to their direct effect on the pathogen, to the appearance of new, resistant strains of Foa [1]. The search for date palm cultivars resistant to Foa remains the best means of combating Bayoud disease. However, only six cultivars producing dates of low quality are resistant to Foa among the 223 cultivars recorded in Morocco [11]. Part of the national date palm sector improvement program deals with the selection and multiplication of genotypes combining resistance to Foa and the production of good quality dates, through genetic crosses between cultivars resistant to Foa and susceptible ones that produce good quality dates [12].

The date palm has developed several defense mechanisms in response to *F. oxysporum* f. sp. *albedinis*, such as the induction of phytoalexin biosynthesis [13], accumulation of caffeoylshikimic acids [14,15], intensification of lignification and the accumulation of cell wall-bound phenolics [16,17,18]. The induction of these mechanisms has been always observed early in development and intensively in resistant cultivars, while it is late and weak in susceptible cultivars. These mechanisms depend on the level of phenylalanine ammonia-lyase activity (PAL), a key enzyme in the phenolic metabolism. The stimulation of PAL activity is related to a natural polysaccharidic elicitor [19]. In addition, PR-proteins such as chitinase and *β*-(1,3)-glucanase have also been reported in date palm in response to infection with *F. oxysporum* f. sp. *albedinis* [20].

To avoid the use of fungicides that are harmful to the environment and human health, biological alternatives have been used to protect and conserve the particular natural environment of oasis palm groves. Among the new biocontrol approaches, the induction of resistance in plants through stimulation of natural defenses is one of the biological control strategies that have shown promising results for the protection of crops in the context of sustainable eco-production [21,22,23,24,25,26]. Natural defense stimulators of plants have been formulated, some of which are based on seaweed polysaccharides and already marketed [21,27,28]. In this context, we recently showed that the alginate extracted from the brown seaweed *Bifurcaria bifurcata* stimulates the natural defenses in date palm roots, in particular by increasing PAL activity and phenolic compound accumulation [29]. Given that finding, this present work sought to confirm the elicitor potential of alginate at the transcriptional level and to evaluate its efficacy against *F. oxysporum* f. sp. *albedinis*. Therefore, we analyzed the expression of genes involved in phenolic metabolism (PAL), oxidative burst (peroxidase: POD, superoxide dismutase: SOD and lipoxygenase: LOX) and PR-proteins (chitinase and *β*-(1,3)-glucanase). The inductor and protector effect of alginate has been compared to a natural defense stimulator formulated from laminarin polysaccharide extracted from the brown algae *Laminaria digitata* and marketed as active substance in Iodus 40^®^ (Goëmar, France).

## 2. Results

### 2.1. Date Palm Gene Expression in Response to Alginate Treatment

#### 2.1.1. PAL Activity and Gene Expression

**Figure 1:**
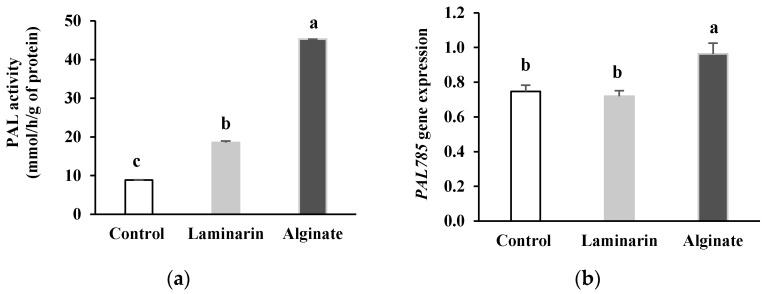
PAL activity (**a**) and relative expression of *PAL785* gene (**b**) in date palm roots pretreated with *Bifurcaria bifurcata* alginate and laminarin. Values assigned the same letter are not significantly different according to Tukey’s HSD test at *p* < 0.001 for (**a**) and at *p* < 0.05 for (**b**).

The treatment of date palm plants with alginate extracted from *B. bifurcata* resulted in a rapid increase in PAL activity within date palm roots and reached almost 5 times it level compared to the control after 24 h following elicitation (Figure 1). Laminarin induced two times the PAL activity observed in the control. However, alginate boosted PAL activity 2.5 times more than laminarin. Likewise, the transcription rate of the *PAL785* gene in alginate-treated plants was significantly higher compared to that in the controls. In addition, plants treated with laminarin presented *PAL785* gene expression that was not significantly different from the control.

#### 2.1.2. Expression Profile of Genes Involved in Oxidative Burst

**Figure 2:**
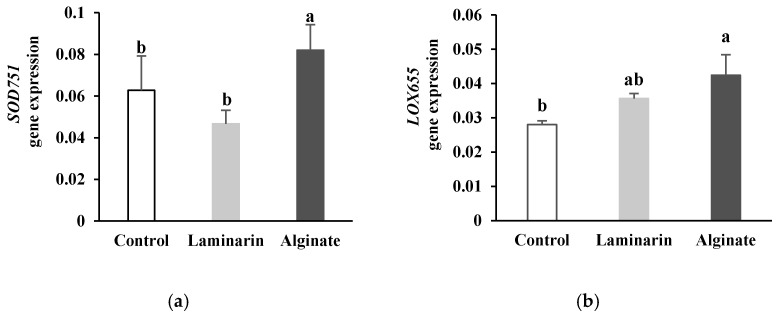

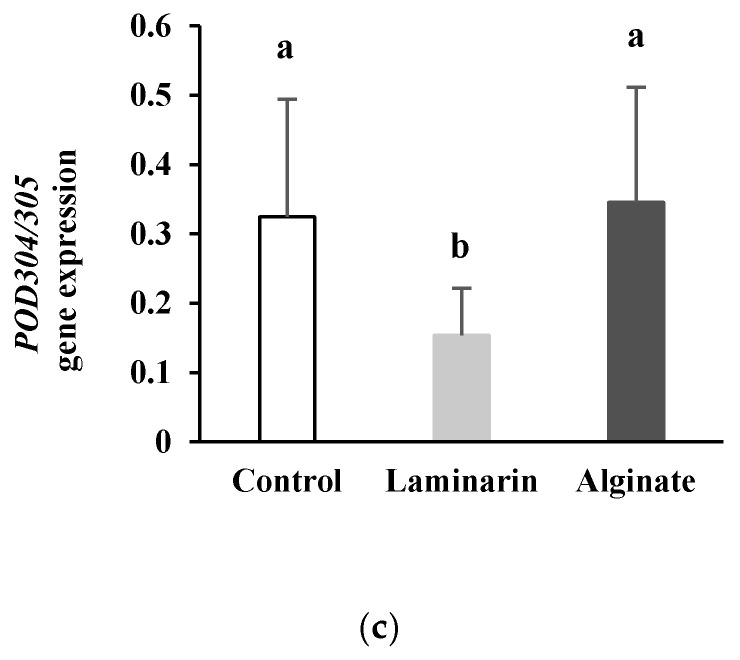
Relative expression of *SOD751* (**a**), *LOX655* (**b**) and *POD304/305* (**c**) genes encoding oxidative burst enzymes (POD: Peroxidase, SOD: Superoxide dismutase and LOX; lipoxygenase) in date palm roots pretreated with *Bifurcaria bifurcata* alginate and laminarin. Values assigned the same letter are not significantly different according to Tukey’s HSD test at *p* < 0.05.

The elicitation of date palm plants by alginate was accompanied in date palm roots after 24 h by an increase in the expression level of major genes involved in oxidative metabolism (POD, SOD, LOX) (Figure 2).

The roots of the alginate-treated plants exhibited significantly higher transcription levels of the *SOD751* (Figure 2a) and *LOX655* (Figure 2b) genes than the levels observed in the control plants (*p* < 0.05), whereas treatment with alginate did not significantly affect gene expression of *POD304/305* (Figure 2c); however, within laminarin-treated plants, *POD304/305* gene expression was repressed while lipoxygenase expression was more strongly induced compared to the control (*p* < 0.05). In addition, alginate induced overexpression of genes involved in oxidative burst, such as *PAL785*, compared to plants treated with laminarin (known as the active ingredient in Iodus 40^®^).

#### 2.1.3. PR-Protein Gene Expression

**Figure 3:**
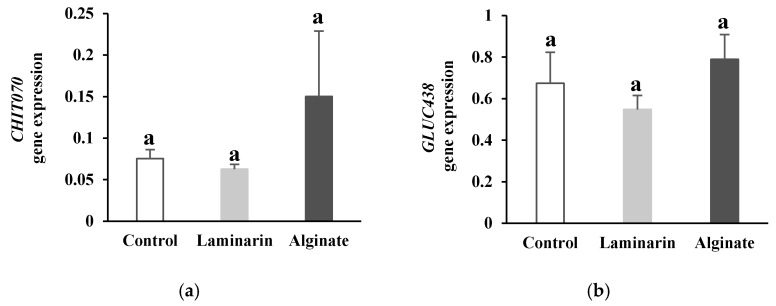
Relative expression of *CHIT070* (**a**) and *GLUC438* (**b**) genes encoding PR-proteins chitinase and *β*-(1,3)-glucanase in date palm roots pretreated with *Bifurcaria bifurcata* alginate and laminarin. Values assigned the same letter are not significantly different according to Tukey’s HSD test at *p* < 0.05.

Unlike genes involved in phenolic metabolism and oxidative burst, alginate did not have a significant effect on the expression of genes encoding PR-protein respectively for chitinase and *β*-(1,3)-glucanase (*CHIT070* and *GLUC438*) (Figure 3). Likewise, the rate of gene transcription of PR-proteins induced by alginate in date palm roots was not significantly different from the rate obtained with laminarin treatment.

### 2.2. Effect of Alginate on Fusarium Wilt Disease

#### 2.2.1. Disease Severity

**Figure 4:**
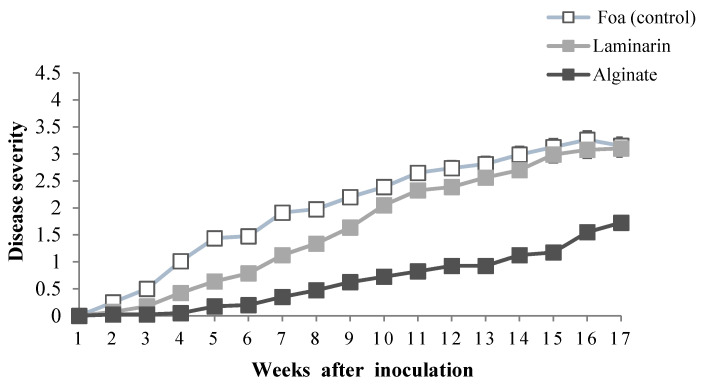
Progress of disease severity in response to elicitation with 1 g·L^−1^ of saccharide solution 1 day before inoculation with *Fusarium oxysporum* f. sp. *albedinis* (Foa).

Inoculation with Foa produced typical symptoms of Fusarium wilt after the 2nd week in inoculated and non-elicited plants (control) (Figure 4). The intensity of symptom progression depended on the saccharide elicitor. In general, all elicited plants developed fewer symptoms compared to the controls. Laminarin-treated date palm developed leaf-refolding symptoms from the 4th week and then progressed with almost the same trend as non-elicited plants. However, plants treated with alginate took almost 7 weeks to develop leaf curl symptoms and progressed slightly compared to control plants and plants elicited by laminarin.

#### 2.2.2. Disease Incidence

**Figure 5:**
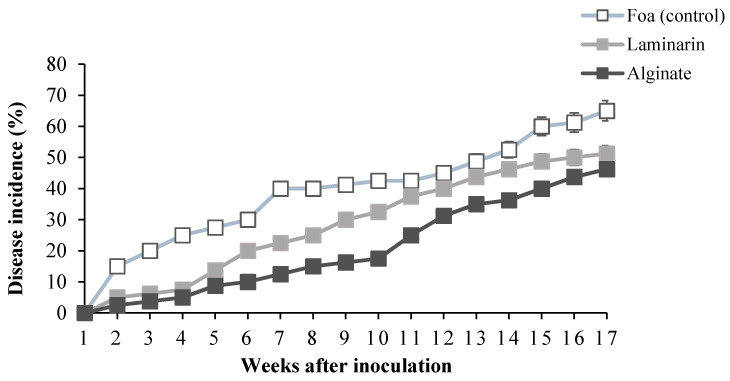
Progress of disease incidence in response to elicitation with 1 g·L^−1^ of saccharide solution 1 day before inoculation with *Fusarium oxysporum* f. sp. *albedinis* (Foa).

The application of alginate from the brown algae *B. bifurcata* significantly reduced the development of fusarium vascular wilt (Figure 5). In the first 10 weeks after inoculation, application of laminarin reduced disease incidence by 10% compared to non-elicited plants (control). However, in plants treated with *B. bifurcata* alginate, the reduction was 25% compared to non-elicited plants. After 3.5 months of inoculation, alginate applied only once reduced the disease incidence by 15% (Table 1).

#### 2.2.3. Effect of Alginate on Date Palm Death Rate

**Figure 6:**
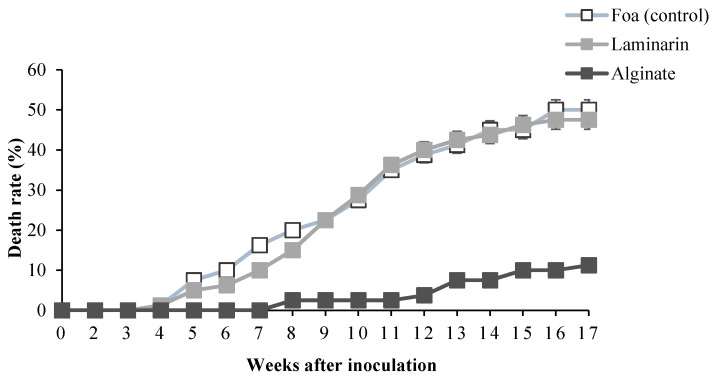
Progress of date palm death rate in response to elicitation with 1 g·L^−1^ of saccharide solution 1 day before inoculation with *Fusarium oxysporum* f. sp. *albedinis* (Foa).

Treatments with alginate of *B. bifurcata* halved plant mortality from the 9th week until the end of the experiment compared to the rate in non-elicited plants (control). However, over 20% of the laminarin-treated plants wilted after 9 weeks of infection with *F. oxysporum* f. sp. *albedinis*, reaching 50% mortality after 3.5 months of experimentation (Figure 6). AUDPC data (Table 1), which summarize the total amount of infection over the 3.5 months of experimentation, show that total infection with *F. oxysporum* f. sp. *albedinis* was significantly elevated (*p* < 0.05) in control plants inoculated with Foa, reaching 40.35% compared to plants inoculated after elicitation with alginate (18.08%). The obtained results (Figure 6, Table 1) show that in date palms, alginate elicited 2 times the resistance against Foa compared to laminarin, known as the active ingredient in Iodus 40^®^.

## 3. Discussion

In order to develop biological alternatives to reduce the incidence of date palm Bayoud disease caused by *F. oxysporum* f. sp. *albedinis*, stimulation of natural plant defenses is currently one of the promising methods for crop protection [25,26]. This biological approach does not exert a direct effect on the pathogen, the origin of a selective pressure that can lead to new and more virulent strains, but stimulates the natural defenses of plants, often leading to resistance of the systemic resistance acquired type [22,30,31,32].

Considering that the defense mechanisms of plants are induced by carbohydrate elicitors [24,33], particularly in the date palm’s defense system against *F. oxysporum* f. sp. *albedinis* [19], the present work aimed at developing a formulation based on polysaccharides from a renewable natural resource, marine algae. Previous works have shown that the ulvan and glucuronan extracted from a green alga, *Ulva lactuca*, stimulate the natural defenses of tomato [31], olive tree [34] and post-harvest apple [35,36]. Likewise, alginate extracted from the brown algae *B. bifurcata*, could also stimulate the natural defenses of tomatoes [37] and date palm [29].

In response to *F. oxysporum* f. sp. *albedinis*, the date palm develops a defense system whose main components derive from the metabolism of phenylpropanoids such as phytoalexins [13], caffeoylshikimic acids [14,15], cell wall-bound phenolics and lignin [16,18]. The induction of the last compounds depends on the level of activity of PAL, a key enzyme in phenylpropanoid metabolism [38]. In a previous work, we showed that elicitation of the date palm by the alginate extracted from *B. bifurcata* at the concentration of 1 g·L^−1^ was accompanied by the stimulation of PAL activity in root with a maximum response 24 h after treatment. In the present work, alginate stimulated PAL activity in date palm roots nearly 5 times more than the level in the control after 24 h. Similarly, the stimulation of PAL activity by alginate was 2.5 times higher than that obtained with the laminarin treatment used as a stimulator of the natural defenses of plants under the name Iodus 40^®^ (Goëmar, France) [39]. As far as the main defense responses can be monitored at the transcriptional level, the analysis of the expression profile of genes involved in defense mechanisms in response to elicitation by alginate in date palm can confirm its inducing effect. In addition, the expression analysis can be used to support the product as a potential candidate in the formulation of natural defense stimulators of plants in general and date palm in particular.

Therefore, we analyzed the expression of six genes involved in the defense system of the date palm (*PAL785*, *POD304/305*, *SOD751*, *LOX655*, *CHIT070* and *GLUC438*). The results obtained showed that the treatment of date palm plants by alginate resulted in the stimulation of *PAL785* gene expression encoding PAL, an enzyme strongly involved in the control of the main defense mechanisms of date palm [19]. The transcription level of the *PAL785* gene in alginate-treated plants was significantly higher than in control plants and even higher compared to plants treated by laminarin, whose level of expression was not significantly different from the control. This result confirms our previous results, which clearly showed the stimulation of date palm defense mechanisms by alginate [29] and thus suggests that alginate could have action at the transcriptional level.

The other defense genes analyzed (*POD304/305*, *SOD751*, *LOX655*) are related to genes involved in the first oxidative events of plant–pathogen interactions resulting from an oxidative burst. Twenty-four hours after alginate treatment, date palm plants exhibited an increase in the expression of genes encoding the main enzymes involved in the oxidative burst in roots (POD, SOD and LOX). *POD304/305*, *SOD751*, *LOX655* genes appeared to be overexpressed in the roots of alginate-elicited plants compared to their expression levels in the control plants, in particular the SOD and LOX genes. On the other hand, such as in the case of the *PAL785* gene, alginate stimulated the expression of the genes involved in the oxidative burst more than did laminarin. Alginate appears to upregulate date palm immunity through the expression of genes involved in natural defenses related to oxidative metabolism, notably the *SOD751* and *LOX655* genes. It is known that oxidative metabolism is one of the main rapid defense responses of the plant, involving the generation of reactive oxygen species (ROS) that include highly reactive reduced molecules such as superoxide anion (O_2_^−^), hydrogen peroxide (H_2_O_2_), the hydroxyl radical (OH^•^) and the hydroperoxyl radical (HO_2_^•^) [40,41]. In addition to their antimicrobial action [42], the intervention of ROS in defense during the early stages of infection is often crucial in plant resistance to pathogens through involvement in various defense components such as the expression of plant defense genes [43,44], induction of hypersensitivity reaction [41], formation of lignin and suberin [45] and the production of oxylipids endowed with signaling functions [46]. In addition, ROS are strongly interconnected with signal molecules inducing expression of defense genes related to hormones such as ethylene, salicylic acid and jasmonic acid [47,48]. Maintaining a healthy and viable biological system results from a balance between the rate of production of ROS and the performance of the antioxidant system [49]. Plants express genes encoding for enzymes involved in the attenuation of cellular damage caused by ROS, including SOD and POD. SOD catalyzes the disproportionation of O_2_^−^ to H_2_O_2_ and O_2_ [50], and POD decomposes H_2_O_2_ into H_2_O and O_2_ [51]. LOX accumulates transiently in response to environmental stimuli and has a key role in defense responses against biotic and abiotic stresses [52,53]. ROS also initiates the hydroperoxidation of polyunsaturated fatty acids producing oxylipins such as jasmonic acid, the role of which as a signal molecule is sometimes crucial [54]. Thus, stimulation of the expression of SOD, POD and LOX genes in date palm by alginate could enhance PAL-dependent defense mechanisms [19].

In addition to phenolic and oxidative metabolisms, PR-protein synthesis was also carried out in response to alginate treatment by monitoring the expression of genes encoding chitinase and *β*-(1,3)-glucanase. PR-proteins represent one of the most commonly induced proteins during plant defense processes and play an important role in plant immunity [55]. Stimulation of chitinase and *β*-(1,3)-glucanase activity was reported in date palm, and cultivars resistant to Bayoud disease showed higher activity levels compared to susceptible cultivars [20]. Our results obtained at the transcriptional level showed that alginate, similarly to laminarin, had no significant effect on expression of the two targeted genes *CHIT070* and *GLUC438*. Analysis of a single gene encoding chitinase and a single gene encoding *β*-(1,3)-glucanase suggested the presence of other genes encoding chitinases and *β*-(1,3)-glucanases that were not taken into consideration in our work and which may explain the differential response between the activity levels [20] and the transcriptional levels of these PR-proteins.

Although laminarin is a sulfated polysaccharide whose biological properties are highly interesting [56,57,58], including as an inducer of resistance against pathogens [21,59,60], alginate (non-sulphated polysaccharide) induces a stronger upregulation of date palm defense gene expression compared to laminarin at low concentration (1 g·L^−1^). This could be linked to the low concentration used in this work (1 g·L^−1^) compared to the one advised in the packaging of Iodus 40^®^ intended for agricultural applications (37 g·L^−1^) [21]. The weak effect of laminarin on date palm defense gene expression could also be explained by differential effects depending on the plant species, since laminarin has been shown to induce natural defenses and resistance in wheat [21] and grapevine [60]. In addition, upstream to this study, we identified several genes coding for the six defense genes analyzed, but we only tested a pair of primers per selected gene encoding the enzymes studied. Laminarin may then stimulate the expression of other genes encoding the enzymes studied. However, the PAL activity believed to be the expression result of all genes was 2.5 times lower compared to that obtained with the alginate treatment. Our results suggest that alginate extracted from *B. bifurcata* used at low concentration better activates the expression of date palm defense genes compared to laminarin, the active ingredient in Iodus 40^®^.

The activation of date palm defense genes by alginate could be linked to an interaction with the main signaling molecules triggering the expression of acquired systemic resistance [61] and induced resistance [62]. It is currently known that plants develop an innate immunity system in response to pathogens, the punctual detection of signaling molecules that constitutes the first step of defense, which consists of a recognition process at the origin of triggered immunity [63,64,65]. The plant can recognize, through recognition receptors anchored to the plant cell surface, (i) non-pathogenic microorganisms through microbe-associated molecular patterns (MAMPs) that are highly conserved molecular traits, (ii) pathogens through pathogen-associated molecular patterns (PAMPs) and (iii) damage-associated molecular patterns (DAMPs) that represent warning signals that indirectly lead to identify pathogens [66,67]. MAMPs, PAMPs and DAMPs were involved in the activation of various signal transduction pathways and defense gene expression [64,68] accompanied by the release of reactive oxygen species [41] and inducing the biosynthesis of signal molecules involved in the expression of defense genes related to hormones such as ethylene, salicylic acid and jasmonic acid [67].

Several saccharide molecules were able to mimic the effect of MAMP, PAMP and DAMP and trigger the natural defenses of plants via signaling molecules [23,24,25,26]. As in the case of laminarin [21,59,60], alginate can induce the expression of defense genes through the pathways of salicylic acid, jasmonic acid or ethylene either as a polymer [69] or oligoalginates [32].

Furthermore, the efficiency of alginate elicitation against date palm Bayoud disease was investigated by the inoculation of seedlings of the sensitive “Jihel” variety of date palm with *F. oxysporum* f. sp. *albedinis* 24 h following elicitation with 1 g·L^−1^ of alginate. The obtained results showed that alginate treatment markedly reduced the extent of vascular Fusarium wilt, through reducing the disease severity and incidence, by three-fold compared to non-elicited plants as well as laminarin-treated plants, leading to an almost 80% reduction in mortality compared to laminarin treatment after 17 weeks. These results highlight the efficiency of the induction of date palm defense gene expression by alginate extracted from *B. bifurcata* against *F. oxysporum* f. sp. *albedinis*, which could be a promising method for sustainable biological control of Bayoud disease.

In summary, our results suggest that *B. bifurcata* alginate acts as an inducer of the expression of the date palm’s natural defense genes at low concentrations compared to laminarin, the active substance in Iodus 40^®^, leading to the highest resistance against *F*. *oxysporum* f. sp. *albedinis*. This work strongly supports the application of alginate as a natural plant defense stimulator and a bioprotector against soil-borne plant pathogens in general and *F. oxysporum* f. sp. *albedinis* in particular. Nonetheless, these results remain to be confirmed on the date palm tree in its natural environment.

## 4. Materials and Methods

### 4.1. Extraction and Purification of Alginate

The alginate fraction was extracted from the brown seaweed *Bifurcaria bifurcata* R. Ross harvested from the Moroccan Atlantic coast of El Jadida city. The pure alginate was produced according to the extraction, purification and characterization techniques described previously [29,70].

### 4.2. Elicitation of Date Palm Natural Defenses

The elicitor effect of alginate was tested on 3-month plants of a susceptible date palm cultivar (Jihel cultivar). Plants were cultivated in a greenhouse at 30 °C, the photoperiod was 16/8 h (day/night), the humidity at 20% and the lighting intensity was 240 µmol/m^2^/s. Elicitation was carried out by soaking the roots in an alginate aqueous solution at 1 g·L^−1^. The optimal concentration was used according to Bouissil et al. (2020b) [29]. Meanwhile, the control plants were pretreated with distilled water. The elicitor capacity of alginate was compared to that of laminarin extracted from *Laminaria digidata* (Sigma-Aldrich, Chimie Sarl, St-Quentin Fallavier, France), described as the active molecule in Iodus40^®^ (Goëmar, France) used at the same concentration of 1 g·L^−1^. The maximum induction of the date palm natural defenses by alginate was observed after 24 h of elicitation [29]. The PAL activity was determined, and the expression of defense genes was evaluated in roots after 24 h. The reported data represent the average of three replicates with three plants per replicate.

### 4.3. Extraction and Determination of PAL Activity

PAL activity was determined according to the method described previously [29]. The date palm roots (250 mg) were crushed in 3 mL of borate buffer (100 mM, pH 8.8) containing 1 mM of EDTA and 5% of insoluble polyvinyl polypyrrolidone (PVPP) (*w*/*w*). After centrifugation at 10,000× *g* for 30 min, the supernatant was analyzed as the enzymatic extract. After incubation for 1 h at room temperature of 30 °C, of the reaction mixture containing 600 mL of the enzymatic extract, 250 mL of L-phenylalanine (20 mM) and 1 mL of borate buffer (100 mM, pH 8.8); the reaction was stopped by adding 100 µL of HCl (6N). PAL activity was determined by measuring the optical density of the trans-cinnamic acid formed at 290 nm. A standard curve was produced with trans-cinnamic acid under the same experimental conditions. The PAL activity was calculated in mmoles of trans-cinnamic acid·h^−1^.mg^−1^ of protein.

### 4.4. Extraction of Total RNA and Synthesis of cDNA

The date palm roots were crushed in liquid nitrogen using a mortar and a pestle. Total RNA was extracted using the NucleoSpin^®^ RNA Plant Kit (Macherey-Nagel, France) according to the manufacturer’s instructions with optimization to the date palm roots, which contain very high concentrations of phenolic compounds and proteins. The crushed roots (280 mg) were added to 40 mg of PVPP and dissolved in a mixture containing 1 mL of extraction buffer RL1 (30–45% of guanidine thiocyanate) and 10 μL of *β*-mercaptoethanol in order to degrade the plasma membrane of root cells. The mixture was then centrifuged at 11,000× *g* for 2 min. The recovered supernatant was filtered and then centrifuged for 1 min at 11,000× *g*. A volume of 500 μL of cold ethanol (70%) was added to the filtrate, homogenized and then loaded in a column retaining the total RNA, then centrifuged for 30 s at 11,000× *g*. After removing the filtrate, 350 μL of membrane desalting buffer (MDB: 1–15% guanidine thiocyanate and 5–20% ethanol) was loaded in the same column and then centrifuged at 11,000× *g* for 1 min to dry the membrane. Genomic DNA in total RNA was removed by digestion with DNase without RNase (Thermo Scientific, France) for 15 min following the manufacturer’s protocol. The RNAs were then eluted with 15 μL of sterile water (RNase-free H_2_O). The amount and purity of total RNA were evaluated using a Thermo Scientific™ NanoDrop™ One Microvolume UV-Vis spectrophotometer and their integrity was verified by 2% agarose gel electrophoresis. The samples were then stored at −80 °C.

In order to be amplified and detected by RT-qPCR, cDNA was synthesized from 200 ng of total RNA using the commercial cDNA synthesis kit (Thermo Scientific Maxima First Strand cDNA Synthesis) according to the manufacturer’s instructions. This kit included the DNAse treatment at the beginning of the protocol in order to degrade any traces of genomic DNA. RNase H treatment (Thermo Scientific 18021-071) was performed after reverse transcription in order to remove residual RNA. The complementary DNA was then stored at −20 °C.

### 4.5. Primer Design and Efficiency

First, genomic sequences of six categories of gene families involved in plant defense mechanisms (phenylalanine ammonia lyase (PAL), peroxidase (POD), superoxide dismutase (SOD), lipoxygenase (LOX), chitinase and *β*-(1,3)-glucanase) were retrieved from the NCBI database (https://www.ncbi.nlm.nih.gov/gene/ (accessed on 13 May 2020)) and analyzed. Six specific primers corresponding to one gene of each gene family were designed using three software packages, MultAlin (http://multalin.toulouse.inra.fr/multalin/ (accessed on 13 May 2020)), Bioedit and Primer3 (http://frodo.wi.mit.edu (accessed on 13 May 2020)). The sequences and characteristics of the primers of the six studied genes are presented in Table 2. A temperature gradient was performed in order to determine the optimum amplification temperature for each pair of primers. Likewise, the primer specificity was verified by the presence of a single amplification peak on the melting curve during RT-qPCR. Finally, the amplification efficiency of each pair of primers was estimated using RT-qPCR amplification on cDNA from a half-dilution range carried out on six points.

### 4.6. Quantitative Real-Time PCR

RNA expression levels of the six genes were analyzed by quantitative real-time PCR (qRT-PCR) carried out on the GENTYANE high throughput genotyping platform (UMR 1095 GDEC, Clermont-Ferrand, France, (http://gentyane.clermont.inra.fr (accessed on 16 October 2021)). The distribution of the 15 µL reaction mix containing 5 ng of cDNA, 0.8 nM gene-specific primers and 7.5 μL of LightCycler^®^ 480 SYBR Green I Master (Roche Diagnostics #04887352001), was carried out using the Microlab Hamilton Star robot in 384-well qPCR plates. The RT-qPCR reaction was carried out using Roche LightCycler^®^ 480 system according to the following program: pre-incubation at 95 °C for 10 min, 45 amplification cycles of 95 °C for 10 s, optimal temperature of the primer for 15 s, and 72 °C for 15 s. The experiment was performed in three biological replicates with three plants per replicate, and all RT-qPCR reactions were performed in two technical replicates per cDNA sample. The relative expression of the analyzed genes was determined according to the 2^−ΔΔCt^ method described previously [72] using the eEF1a gene [71] as an internal reference for normalization.

### 4.7. Fungal Material

A purified strain of the fungus *Fusarium oxysporum* f. sp. *albedinis* (Foa), isolated from palm rachis affected by Bayoud disease was transplanted on potato dextrose agar (PDA) medium and then incubated in the dark and at 25 °C for 15 days. The inoculum was supplied as a conidial suspension by washing the 15-day-old Foa culture with sterile distilled water, then it was collected and filtered twice to remove mycelial fragments. The number of conidia was determined using Malassez cell, and the inoculum concentration was adjusted with sterile distilled water to 10^6^ conidia·mL^−1^ [73].

### 4.8. Inoculation Test

The roots of the 3-month-old plants were soaked in alginate and laminarin solutions at 1 g·L^−1^ on a sampling of 3 blocks/treatment with 14 plants per block. In coextending, control plants were elicited with distilled water. After 24 h, the saccharide solutions were removed, and the plants were inoculated at the radicle level with 20 mL (10^6^ conidia·mL^−1^) of Foa inoculum; the control plants were inoculated with sterile distilled water. The symptoms of Foa infection on each plant were recorded every 4 days after inoculation for 3.5 months.

### 4.9. Disease Evaluation

Symptoms of *Fusarium oxysporum* f. sp. *albedinis* (Foa) infection were estimated by calculating for each plant the disease severity and incidence as well as the death rate every 4 days after inoculation with Foa inoculum for 3.5 months. These parameters were determined according to the formulas described previously [74].

Therewith, the intensity of the disease over time expressed by the area under the disease progress curve (AUDPC) was calculated for each block using the maximum value of severity (FMS) potentially reached in the assessment period according to the Campbell and Madden method [75].

### 4.10. Statistical Analysis

The results were analyzed using a randomized factorial design with three replicates and three plants per replicate for the qRT-PCR test, and with three blocks of 14 plants per block for the protection test. Data analysis was carried out by one-way analysis of variance (ANOVA) using Tukey’s test at *p* < 0.05 for multiple comparisons using SPSS software version 20.0.

## 5. Conclusions

The obtained results showed clearly that *B. bifurcata* alginate at low concentration (i) induces the defense mechanisms of date palm, (ii) stimulates expression of the genes involved in phenolic metabolism (PAL) and burst oxidation (LOX and SOD) and (iii) greatly improves the resistance of date palm to Foa. In addition, the comparison of alginate with laminarin, the active ingredient in Iodus 40^®^, showed that a single application of alginate at a low dose was 2.5 times more effective in terms of induction of defense mechanisms and improvement of resistance to Foa compared to the application of laminarin at the same dose. These results open up promising prospects for a new biological control strategy based on the stimulation of plant defenses.

## Figures and Tables

**Table 1:** Incidence, death, FMS and AUDPC of vascular fusarium wilt of date palm plants calculated after 3.5 months of inoculation with Foa.

	Disease Incidence (%)	PDP ^1^ (%)	FMS ^2^	AUDPC ^3^ (%)
**Foa**	65 ^a^	50 ^a^	2.11 ^a^	40.35 ^a^
**Laminarin**	52.5 ^ab^	47.5 ^a^	1.71 ^b^	32.11 ^b^
**Alginate**	47.5 ^b^	12.5 ^b^	0.68 ^c^	13.86 ^c^

Means within column followed by the same letter are not significantly different at *p* < 0.05. ^1^ PDP = percentage of dead plants. ^2^ FMS = final mean severity of symptoms 3.5 months after inoculation. ^3^ AUDPC = area under the disease progress curve with reference to the maximum value potentially reached over the assessment period.

**Table 2:** Primer pairs designed on genes encoding phenylalanine ammonia lyase (PAL), lipoxygenase (LOX), peroxidase (POD), superoxide dismutase (SOD), *β*-(1,3)-glucanase (GLUC) and chitinase (CHIT).

Genes	Primers
*PAL785*	F:5′-GGGATTGGAAAAGTCTGCAG-3′ R:5′-ACCACATGTACCCATAGCC-3′
*LOX655*	F: 5′-AGGCCTCCAACCAATACAG-3′ R: 5′-TCGTGGAAGGCCTTGAAGT-3′
*POD304/305*	F: 5′-TTCTCTCAGGTGGGCATACAA-3′ R: 5′-AAAGCTCCCAGGATCCATTT-3′
*SOD751*	F: 5′-TCCATGCCGCCCAGGTCT-3′ R: 5′-CATCTAACCTATTCGCCTTG-3′
*GLUC438*	F: 5′-CGGCCCATCAGACTCCAA-3′ R: 5′-GCCTCAATTACCAATTTTGCA-3′
*CHIT070*	F: 5′-CCATGAAACAACTGGTGGG-3′ R: 5′-TTTACCAGCCGGTCCATAG-3′
*eEF1a* [71]	F: 5′-GATCCCTTCCTACACTCGAATCC-3′ R: 5′-TCCTTTCCCATTGGTATTTGCT-3′
